# The rationale and cost-effectiveness of a confirmatory mapping tool for lymphatic filariasis: Examples from Ethiopia and Tanzania

**DOI:** 10.1371/journal.pntd.0005944

**Published:** 2017-10-04

**Authors:** Katherine M. Gass, Heven Sime, Upendo J. Mwingira, Andreas Nshala, Maria Chikawe, Sonia Pelletreau, Kira A. Barbre, Michael S. Deming, Maria P. Rebollo

**Affiliations:** 1 Neglected Tropical Disease Support Center, Task Force for Global Health, Atlanta, United States of America; 2 Ethiopian Public Health Institute, Addis Ababa, Ethiopia; 3 Neglected Tropical Disease Control Program, Ministry of Health and Social Welfare, Dar es Salaam, Tanzania; 4 IMA World Health Tanzania, Dar es Salaam, Tanzania; 5 Children’s Investment Fund Foundation, London, United Kingdom; 6 Consultant, Neglected Tropical Diseases Support Center, Task Force for Global Health, Atlanta, United States of America; Case Western Reserve University School of Medicine, UNITED STATES

## Abstract

Endemicity mapping is required to determining whether a district requires mass drug administration (MDA). Current guidelines for mapping LF require that two sites be selected per district and within each site a convenience sample of 100 adults be tested for antigenemia or microfilaremia. One or more confirmed positive tests in either site is interpreted as an indicator of potential transmission, prompting MDA at the district-level. While this mapping strategy has worked well in high-prevalence settings, imperfect diagnostics and the transmission potential of a single positive adult have raised concerns about the strategy’s use in low-prevalence settings. In response to these limitations, a statistically rigorous confirmatory mapping strategy was designed as a complement to the current strategy when LF endemicity is uncertain. Under the new strategy, schools are selected by either systematic or cluster sampling, depending on population size, and within each selected school, children 9–14 years are sampled systematically. All selected children are tested and the number of positive results is compared against a critical value to determine, with known probabilities of error, whether the average prevalence of LF infection is likely below a threshold of 2%. This confirmatory mapping strategy was applied to 45 districts in Ethiopia and 10 in Tanzania, where initial mapping results were considered uncertain. In 42 Ethiopian districts, and all 10 of the Tanzanian districts, the number of antigenemic children was below the critical cutoff, suggesting that these districts do not require MDA. Only three Ethiopian districts exceeded the critical cutoff of positive results. Whereas the current World Health Organization guidelines would have recommended MDA in all 55 districts, the present results suggest that only three of these districts requires MDA. By avoiding unnecessary MDA in 52 districts, the confirmatory mapping strategy is estimated to have saved a total of $9,293,219.

## Author summary

Mapping is used by lymphatic filariasis (LF) elimination programs to determine if mass drug administration (MDA) is required. The current mapping approach, designed to be simple and practical, has worked well in high-prevalence settings but concerns about its reliability in low-prevalence settings have been raised. To address these concerns, a confirmatory mapping strategy was developed that utilizes probability-based sampling of school attending children to determine if the prevalence of LF antigenemia is below a 2% threshold. The confirmatory mapping strategy was implemented in 45 districts in Ethiopia and 10 in Tanzania where the need for MDA was uncertain. In 52 of the 55 districts, the number of LF antigen-positive children identified by the confirmatory mapping strategy was below the predetermined threshold and MDA was deemed unnecessary, while in three districts the number of positive children exceeded the threshold, suggesting that MDA is required. The use of this mapping strategy, to confirm whether MDA is required, is estimated to have saved the Ethiopian and Tanzanian programs $9,293,219 by avoiding unnecessary MDA in 52 districts.

## Introduction

Endemicity mapping is the essential first step for countries striving to eliminate neglected tropical diseases. For lymphatic filariasis (LF), a parasitic disease targeted by the World Health Organization (WHO) for elimination as a public health problem by 2020 [1], endemicity mapping (or ‘mapping’) is conducted at the district level following standard guidelines to determine whether the disease is endemic. In districts found to be endemic the entire at risk population is treated with preventive chemotherapy delivered through mass drug administration (MDA). To date, tremendous progress towards global elimination of LF has been made. By 2015, of the 73 endemic countries, 18 had entered post-MDA surveillance, 25 countries had achieved 100% geographic coverage, 20 were in the process of scaling up MDA to all endemic districts and only 10 countries had yet to start MDA [2].

The current WHO strategy for mapping LF is meant as a practical approach to quickly and easily identify districts where active transmission is occurring. In the African region, two sites (*e*.*g*., villages) considered to be at higher risk than other areas are purposefully selected, based on the presence of persons with chronic morbidity, high mosquito densities or other permissive factors, and within each site a convenience sample of 50–100 people is tested for LF. Though there is some regional variation in the age group sampled, in Africa, where the majority of the mapping gap remains [3], sampling is limited to adults >15 years old [4]. In areas where *W*. *bancrofti* is possibly endemic, LF mapping is conducted using either an immunochromatographic test (ICT) or filariasis test strip (FTS) to measure filarial antigenaemia [5]. Where *Brugia spp*. are possibly endemic, mapping requires blood films to detect microfilaremia. According to WHO guidelines, if the prevalence of microfilaremia or antigenaemia is ≥1% in either of the two sites, the district is classified as endemic and MDA is required [6].

This WHO mapping strategy has been used successfully to map thousands of districts, helping countries to rapidly scale up their LF elimination programs. With its low threshold–a 1% prevalence essentially equates to finding one or more positive individuals–the current strategy favorsprogrammatic action in high prevalence settings; however, in low prevalence settings this mapping approach has several limitations.

Where the prevalence is low, there is often limited information about LF transmission, making it hard to identify sites where the risk of transmission is the greatest. In the absence of detailed clinical information, and given the focality of LF, the reliance on two purposefully selected sites in a district may be insufficient to rule out ongoing transmission where the prevalence is low. Secondly, in low prevalence settings it is hard to know if antigen-positive adults are indicative of active transmission, a reflection of earlier infection, or the introduction of individuals from endemic areas, due to ever-increasing population mobility. Finally, the diagnostics used to detect filarial antigen have imperfect specificity [7], which calls into question the significance of a single positive result. Furthermore, these tests have been shown to share some cross-reactivity with individuals harboring high intensity *Loa loa* infections [8].

Given these limitations, when the current mapping strategy results in the detection of a single antigen-positive adult, the implications for ongoing transmission are uncertain. Indeed, some NTD program managers have been hesitant to base the decision to start resource-intensive MDA on such borderline mapping results. Nonetheless, in order to reach the ultimate goal of elimination, countries must be able to determine the LF endemicity status of each district so that appropriate action can be taken.

To provide greater confidence in the decision to start or forego MDA when the initial mapping results are uncertain, an LF ‘confirmatory mapping’ tool has been developed. The confirmatory mapping tool utilizes cluster sampling of school-attending children and is meant to provide a statistically rigorous tool for programmatic decision-making. This tool was recently piloted in 45 districts of Ethiopia, where initial mapping surveys found only one antigen-positive result per district, and in 10 districts in Tanzania, where endemicity was also uncertain due to the amount of time that had passed since the initial mapping and independent studies suggesting little or no transmission in the area.

In this manuscript, we provide a detailed description of the methodology underlying the confirmatory mapping tool. We present summary findings from the first two pilots of this strategy in Ethiopia and Tanzania, an analysis of the strategy’s cost effectiveness, and the results from two small comparison studies using both the standard mapping strategy and the confirmatory mapping approach.

## Materials and methods

### Ethics statement

Ethical clearance from the local institutional review boards was obtained in advance of each study. In Ethiopia, ethical clearance was received from the Ethiopian Public Health Institute. In Tanzania, the National Medical Research Institute provided clearance. All participation in the survey was voluntary. Permission to conduct the survey was obtained from the directors of the selected schools and a letter to parents explaining the study was sent home with students during the days leading up to the study. Only children with written consent forms signed by their parent or guardian were allowed to participate in the study. There were no adult participants in this study. All children were provided with their test results and positive children were referred to health authorities. Both the Ethiopian and Tanzanian national LF programs were committed to providing treatment with ivermectin and albendazole through MDA according to WHO guidelines. If a district did not qualify for LF MDA based on the confirmatory mapping results, any antigenemic children identified through the survey received individual treatment.

### Target population

Primary schools, which includes all public, private or confessional schools, make up the primary sampling units for the confirmatory mapping tool, due to the logistical advantages schools offer over community-based sampling [9]. Children in grades 4–8 of primary school, which typically corresponds to ages 9–14 years, were targeted for inclusion in the survey. The decision to target older children rather than 6–7 year olds, as in the Transmission Assessment Survey, was based on a desire to improve the chances of detecting infected individuals with the survey. In treatment-naïve settings, older children have a longer period of potential exposure to infection and previous studies suggest that infection in older children is representative of infection in the population as a whole [10,11].

### Sampling strategy

Due to the wide range foreseen for district sizes, two sampling strategies were proposed. In districts with fewer than 40 primary schools, systematic element sampling was used, whereby all schools in the district were visited and a set fraction of students in the targeted grades were included, after adjusting for the expected non-response rate. The same sampling fraction (*f*) was used in each school, resulting in an equal probability of selection for each student in the district (Eq 1).

$$P\left(child_{ij}\right)=f=\frac{target\;sample\;size}{(\sum children_{i})\left(1-nonresponse\;rate^{\prime }\right)}$$ (1)

In larger districts with at least 40 schools, cluster sampling was recommended, whereby 30 schools were selected from a sampling frame that included all primary schools in the district, using sampling with probability proportionate to estimated size. To maintain an equal probability of selection, an independent sampling fraction was calculated for each selected school, based on the expected school enrollment (*school*_*i*_), expected non-response rate, and the target sample size per school (Eq 2).

$$P\left(child_{ij}\right)=30*\frac{school_{i}}{\sum school_{i}}*\frac{target\;school\;sample\;size}{school_{i}*\left(1-nonresponse\right)}=\frac{30*target\;school\;sample\;size}{\left(\sum school_{i}\right)*\left(1-nonresponse\right)}$$ (2)

When the selected school was small and had fewer students expected in the targeted grades than the required sample size, the school was merged with the next school on the list during the first stage of sampling. If one of these merged schools was selected, a sampling fraction for the combined size of the two schools was calculated and the survey team would visit both schools and apply the same sampling fraction in order to reach the desired sample size.

### Sample size

The sample size and decision rules for this survey are based on the null hypothesis that the average prevalence of antigenemia in older school children is ≥2%. A hypothesis test is constructed using the hypergeometric distribution to calculate the probability of finding no more than *d* antigen-positive children in a sample of *n* target-grade children, drawn from a total survey population of *N* such children. Districts in which the number of children testing positive is less than or equal to the critical cutoff, *d*, are said to “pass” the survey and are considered not in need of MDA (i.e. reject the null hypothesis). Conversely, districts in which the number of positive children is greater than the critical cutoff fail to reject the null hypothesis and are considered endemic and in need of MDA. The critical cutoff, *d*, was determined based on <6% risk of Type I error (e.g., the risk of falsely concluding that the prevalence is <2%) and power of approximately 35% of rejecting the null hypothesis when prevalence is 1.0% (half of the threshold). The actual ranges of Type 1 error (α) and power for each sample size are shown in Table 1. The designation of such a low power makes it harder for non-endemic districts (i.e., those with a true antigenemia prevalence <2%) to pass the survey; however, this was deemed advantageous because it is a more conservative approach and biases the tool in favor of starting MDA. The low power of this confirmatory mapping tool, compared to a survey such as the LF Transmission Assessment Survey, which has 75% power when the true prevalence is half the threshold, has the added advantage of dramatically reducing the sample size. Finally, in order to account for the potential clustering of cases by school, the sample size of the cluster-based surveys was multiplied by the estimated design effect of 1.5. The resulting sample sizes and cutoffs, referred to jointly as the ‘decision rules’ for the survey, are shown in Table 1.

**Table 1:** Decision rules for confirmatory mapping surveys.

Population Surveyed (N)^φ^:	Systematic Sample (for districts with <40 schools)				Cluster Survey (for districts with ≥40 schools)	
	Critical Cutoff (d)	Sample Size (n)	Range of α^§^	Power^ѱ^	Critical Cutoff (d)	Sample Size (n)
≥2,000	2	320	3.3%–4.5%	35.7%–37.9%	3	480
1,000–1,999	2	300	3.4%–5.3%	38.2%–44%	3	450
750–999	1	220	3.8%–5.5%	34.3%–37.6%	NA	NA
500–749	1	210	3.4%–6.3%	30.2%–37.1%	NA	NA
<500	0.02*N	Census (N)			NA	NA

^φ^Refers to the size of the entire population of children in the target age group living in the survey area.

^§^ Type 1 error values (α) for the range of population sizes, calculated using the hypergeometric distribution and apply to both systematic and cluster sampling settings.

^ѱ^Power calculations for the range of population sizes, assuming that the true prevalence is 1% (half the threshold) and calculated by the hypergeometric distribution; the power applies to both systematic and cluster sampling settings.

To operationalize the sampling strategy, an Excel-based Confirmatory Mapping Survey Builder tool (http://www.ntdsupport.org/resources/confirmatory-mapping-survey-builder) was created and used by the field teams to select the primary sampling units and generate the sampling lists for each school.

### Testing

Approximately 160ul of whole blood was taken from each participant via finger prick. One hundred microliters were used to assess the presence of *Wuchereria bancrofti* circulating filarial antigen using the immunochromatographic card test (ICT; Alere, Scarborough, ME, USA). Any child testing positive by ICT was retested to confirm the result. The remaining 60ul of whole blood was placed onto filter paper and frozen for future laboratory-based antibody testing.

### Site selection

**Fig 1:**
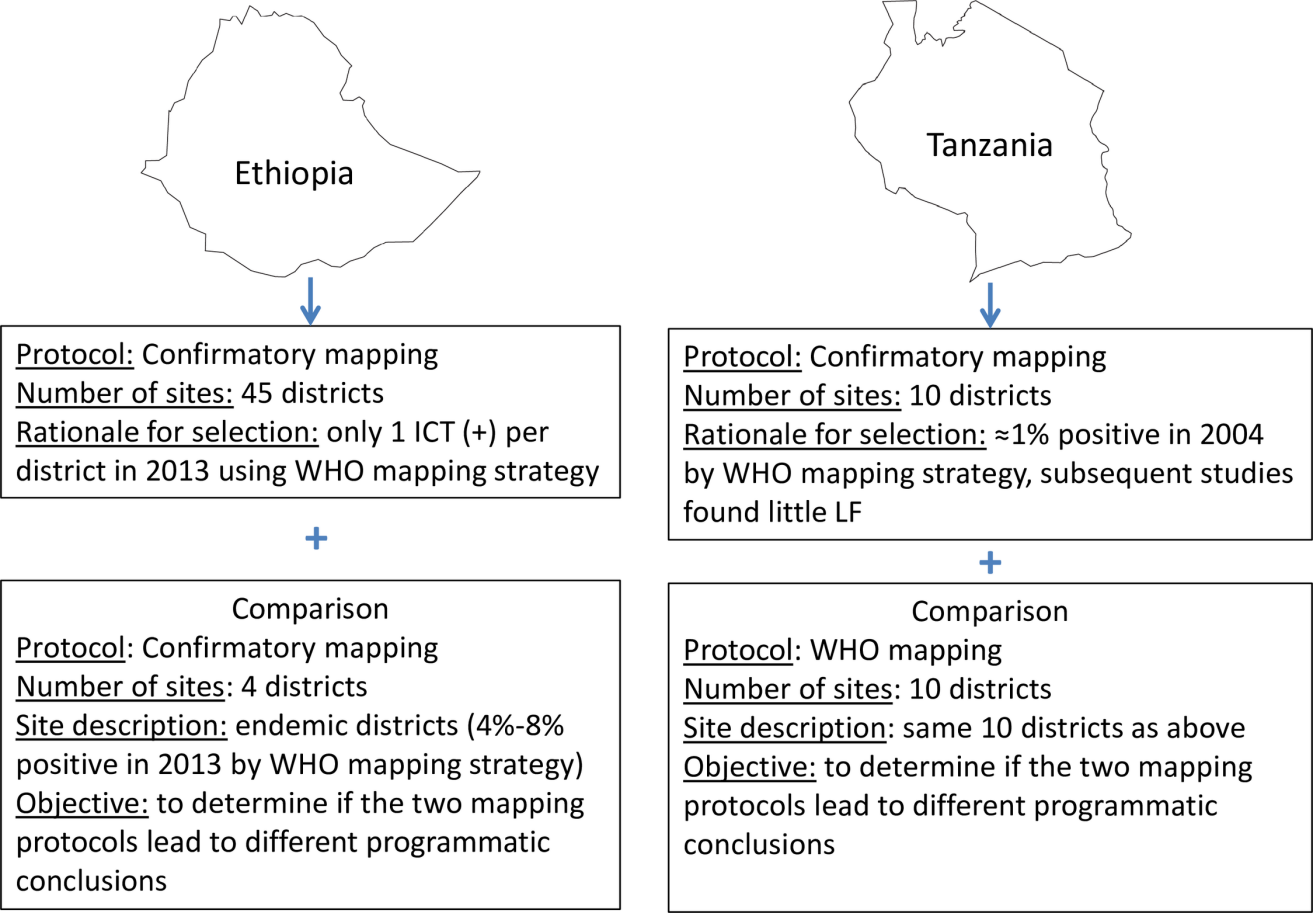
Schematic showing the study designs in Ethiopia and Tanzania.

In Ethiopia, a nationwide mapping exercise, undertaken in 2013, identified 45 districts (locally referred to as *woredas*) with only one ICT-positive individual [12]. Due to the uncertainty regarding the significance of a single antigen-positive adult, the confirmatory mapping tool was applied in all 45 of these districts. In addition, four districts that were declared endemic by the 2013 mapping exercise, with ICT positivity ranging from 4%–8%, were included to compare the standard WHO protocol with the confirmatory mapping strategy and see if both protocols result in a similar ‘endemic’ classification (Fig 1). All districts were reportedly treatment naïve for ivermectin at the time of selection. The confirmatory mapping in Ethiopia took place in two phases: phase 1 took place December, 2014 –January, 2015 and phase two took place December, 2015 –March, 2016.

In Tanzania, the national LF program conducted initial mapping for LF from 1999 to 2004 using the standard WHO protocol and found several districts with ≈1% prevalence of mf. However, subsequent investigations in these same districts by two different research groups found little to no LF, which, combined with the mass distribution of long lasting insecticidal nets and indoor residual spraying for malaria, called into question the need for MDA. Of 63 such districts where LF endemicity was considered uncertain, 10 were selected for confirmatory mapping in 2015 (the remaining 53 districts were mapped at a later date), all of which were ivermectin naïve at the time of selection. Children attended schools in their same community of residence. To enable direct comparison between results of the standard WHO mapping protocol and those of the confirmatory mapping protocol, in each of the initial 10 Tanzanian districts, two communities with the highest suspected risk of ongoing LF transmission were selected (Fig 1). In each purposively selected community, the standard WHO protocol was applied, whereby a convenience sample of 100 individuals ≥ 15 years were tested for LF antigen, for a total sample size of 200 additional individuals per district.

### Cost-effectiveness analysis

To calculate the cost-effectiveness of the confirmatory mapping tool, the actual costs of conducting the confirmatory mapping exercise in all districts were subtracted from the costs averted by avoiding treatment of districts found by confirmatory mapping to be non-endemic for LF. In accordance with current WHO guidelines for LF, the cost of conducting a successful LF elimination program in a district include, at minimum, the costs associated with conducting five rounds of MDA at 65% coverage, two sentinel and spot check assessments, and three transmission assessment surveys [13]. Because treatment costs vary according to district population, we estimated district population using a database maintained by the WHO AFRO Region. A detailed explanation of how the costs for each of these components were estimated is presented as supplementary material (S1 Appendix).

## Results

The confirmatory mapping strategy was conducted in 55 districts, 45 in Ethiopia and 10 in Tanzania (Table 2). A total of 22,614 children in grades 4–8 were tested from 1483 schools. Systematic sampling of schools was called for in 18 smaller Ethiopian districts, while cluster sampling was utilized in the remaining 27 Ethiopian districts and all 10 Tanzanian districts. Twenty-eight ICT positive children were detected from 9 of the 45 districts in Ethiopia; in three of these districts the number of ICT-positive children exceeded the critical cutoff, meaning that the district “failed to pass” the confirmatory mapping assessment and should be considered endemic and in need of MDA. The remaining 42 districts “passed” the confirmatory mapping, meaning that the number of positive individuals was equal to or below the critical cutoff, and were declared not in need of MDA. In Tanzania, only one ICT positive individual was confirmed (two additional children tested positive but a confirmatory test found both to be negative). All 10 districts in Tanzania passed the confirmatory mapping assessment and were declared not in need of MDA for LF. The design effect for the surveys where cluster sampling was used, and which had at least one positive result, ranged from 0.9–5.3, with the majority of the design effects falling between 1.0–2.0. The data used to generate these results is provided in the supplementary information files (S1–S3 Datas).

**Table 2:** Summary of confirmatory mapping tool results by district from Ethiopia and Tanzania, 2015.

Country	Region	District	Survey Design^§^	Schools Sampled	Children Tested	ICT Positive (%)	Design Effect^†^	Survey Result	Mapping Decision
Ethiopia	Afar	Erebti	Systematic	10	269	0	NA	Pass	No MDA
Ethiopia	Amhara	Aneded	Cluster	24	375	0	-	Pass	No MDA
Ethiopia	Amhara	Baso-Liben	Cluster	30	467	0	-	Pass	No MDA
Ethiopia	Amhara	Dangla Zuria	Cluster	29	451	0	-	Pass	No MDA
Ethiopia	Amhara	Ebinat	Cluster	28	447	0	-	Pass	No MDA
Ethiopia	Amhara	Efrata- Gidim	Cluster	30	459	0	-	Pass	No MDA
Ethiopia	Amhara	Fogera	Cluster	30	477	0	-	Pass	No MDA
Ethiopia	Amhara	Guangua	Cluster	30	502	0	-	Pass	No MDA
Ethiopia	Amhara	Inarj-Inawuga	Cluster	29	459	0	-	Pass	No MDA
Ethiopia	Amhara	Jabi_Tahnan	Cluster	29	459	0	-	Pass	No MDA
Ethiopia	Amhara	Merahibete	Systematic	34	522	0	NA	Pass	No MDA
Ethiopia	Amhara	Moret Jiru	Systematic	38	446	0	NA	Pass	No MDA
Ethiopia	Amhara	Semada	Cluster	27	423	4 (1.0%)	1.4	Fail	MDA Required
Ethiopia	Amhara	Tach Gaynt	Cluster	30	461	5 (1.1%)	2.1	Fail	MDA Required
Ethiopia	Harari	Aboker	Systematic	5	194	0	NA	Pass	No MDA
Ethiopia	Oromia	Abbaay Cooman	Systematic	27	332	0	NA	Pass	No MDA
Ethiopia	Oromia	Abbee Dangoorooo	Systematic	31	320	0	NA	Pass	No MDA
Ethiopia	Oromia	Adama	Cluster	30	484	0	-	Pass	No MDA
Ethiopia	Oromia	Amboo	Systematic	8	127	0	NA	Pass	No MDA
Ethiopia	Oromia	Bule-Hora	Cluster	31	471	2 (0.4%)	0.9	Pass	No MDA
Ethiopia	Oromia	Cooraa Botor	Cluster	30	477	1 (0.2%)	1	Pass	No MDA
Ethiopia	Oromia	Daawoo	Systematic	27	379	0	NA	Pass	No MDA
Ethiopia	Oromia	G-Ayana	Cluster	28	472	2 (0.4%)	1	Pass	No MDA
Ethiopia	Oromia	Goro	Systematic	26	328	0	NA	Pass	No MDA
Ethiopia	Oromia	Gura Dhaamolee	Systematic	23	237	0	NA	Pass	No MDA
Ethiopia	Oromia	Kokkossaa	Cluster	31	475	0	-	Pass	No MDA
Ethiopia	Oromia	Miyoo	Systematic	19	268	1 (0.4%)	NA	Pass	No MDA
Ethiopia	Oromia	Qoree	Cluster	30	541	0	-	Pass	No MDA
Ethiopia	Oromia	Sawweena	Systematic	39	408	0	NA	Pass	No MDA
Ethiopia	Oromia	Sibu-Sire	Cluster	30	468	0	-	Pass	No MDA
Ethiopia	Oromia	Sululta	Cluster	27	447	0	-	Pass	No MDA
Ethiopia	Oromia	Wondoo	Systematic	20	311	0	NA	Pass	No MDA
Ethiopia	Oromia	Xanna	Systematic	32	332	0	NA	Pass	No MDA
Ethiopia	Oromia	Yaaballo	Systematic	7	115	0	NA	Pass	No MDA
Ethiopia	SNNP	Debub Ari	Cluster	30	478	10 (2.1%)	5.3	Fail	MDA Required
Ethiopia	SNNPR	Arbegona	Cluster	30	478	0	-	Pass	No MDA
Ethiopia	SNNPR	Bensa	Cluster	30	631	0	-	Pass	No MDA
Ethiopia	SNNPR	Demba Gofa	Cluster	30	481	0	-	Pass	No MDA
Ethiopia	SNNPR	Hulbareg	Systematic	25	288	0	NA	Pass	No MDA
Ethiopia	SNNPR	Kebena	Systematic	23	345	0	NA	Pass	No MDA
Ethiopia	SNNPR	Sawla	Systematic	5	281	0	NA	Pass	No MDA
Ethiopia	Tigray	Adwa (Rural)	Cluster	30	463	2 (0.4%)	1	Pass	No MDA
Ethiopia	Tigray	Atsbi Wenberta	Cluster	30	463	0	-	Pass	No MDA
Ethiopia	Tigray	Gulomekheda	Cluster	29	463	1 (0.2%)	1	Pass	No MDA
Ethiopia	Tigray	Hawzen	Cluster	30	480	0	-	Pass	No MDA
**Ethiopia Total**				1,191	18,254	28			
Tanzania	Arusha	Meru	Cluster	29	460	0	-	Pass	No MDA
Tanzania	Arusha	Monduli DC	Cluster	30	358	0	-	Pass	No MDA
Tanzania	Kagera	Karangwe DC	Cluster	30	453	0	-	Pass	No MDA
Tanzania	Kagera	Muleba DC	Cluster	24	304	0	-	Pass	No MDA
Tanzania	Kilimanjaro	Moshi DC	Cluster	30	481	0	-	Pass	No MDA
Tanzania	Kilimanjaro	Moshi MC	Cluster	30	463	1 (0.2%)	1	Pass	No MDA
Tanzania	Kilimanjaro	Same DC	Cluster	30	477	0	-	Pass	No MDA
Tanzania	Kilimanjaro	Siha	Cluster	29	451	0	-	Pass	No MDA
Tanzania	Mara	Musoma	Cluster	30	439	0	-	Pass	No MDA
Tanzania	Simiyu	Bariadi	Cluster	30	474	0	-	Pass	No MDA
**Tanzania Total**				292	4,360	1			
**TOTAL**				**1,483**	**22,614**	**29**			

^**†**^The design effect estimates the degree to which the cluster survey design increases the sample variance, relative to a simple random sample and will be incalculable (“-“) in districts where all individuals are negative; this calculation of the design effect does not apply to systematic survey designs (“NA” = not applicable).

^§^A systematic survey design implies that all schools in the district were included in the sample and was employed in smaller districts (i.e. those with ≤40 schools total); under a cluster survey design only 30 schools were visited.

The total cost of conducting the confirmatory mapping surveys across the 55 districts was $451,936, with an average cost of $8,217 per district (Table 3). The average cost per survey was less in Ethiopia compared with Tanzania ($7,910 compared with $9,599). The 52 districts that passed the confirmatory mapping assessment represent 8.1 million people no longer in need of MDA and $9.7 million in adverted costs due to the MDA and accompanying monitoring and evaluation that are no longer necessary. This translates into an estimated savings of $9.2 million ($5.7 million in Ethiopia and $3.5 million in Tanzania), after accounting for the cost of all 55 confirmatory mapping surveys and the costs associated with MDA in the 3 endemic districts.

**Table 3:** Cost savings resulting from confirmatory mapping in Ethiopia and Tanzania, calculated by comparing the costs of the mapping surveys with the averted costs for districts that passed and did not required MDA treatment.

Country	Districts mapped	Total cost of mapping*	Average Mapping cost/district	Districts passed	Estimated total pop of passing districts	Averted costs for MDA^†^	Averted cost for spot check/sentinel site testing^‡^	Averted cost for TAS^§^	Total averted costs	Cost savings
**Ethiopia**	45	$355,950	$7,910	42	5,378,528	$4,544,856	$174,720	$1,381,879	$6,101,455	$5,745,505
**Tanzania**	10	$95,985	$9,598	10	2,721,896	$3,273,080	$41,600	$329,019	$3,643,699	$3,547,714
**TOTAL**	**55**	**$451,935**	**$8,217**^**φ**^	**52**	**8,100,424**	**$7,817,936**	**$216,320**	**$1,710,898**	**$9,745,154**	**$9,293,219**

*Total cost of mapping includes both field costs and cost of 500 ICT cards per district (cost of $2.75 per ICT card).

^†^This assumes five rounds of MDA at 65% coverage with a cost of $0.26 per dose in Ethiopia and $0.37 per dose in Tanzania.

^‡^This assumes 2 rounds of sentinel/spot check site testing with 600 participants tested with FTS each round at a cost of $1.80 per FTS. To account for other associated costs, an additional $1,000 per district was added.

^§^This assumes three rounds of TAS at a cost of $24,900 per TAS per evaluation unit. We assumed two districts per evaluation unit, bringing the cost of each round of TAS per district to $12,450.

^φ^Average cost is weighted by the number of districts.

In Ethiopia, the confirmatory mapping protocol was implemented in four additional endemic districts (according to the WHO mapping surveys conducted in 2013). Systematic sampling was used to visit children from all schools in three of these districts, while cluster sampling was used in the fourth (Table 4). In two of the districts (Boneya Bushe and Haro-Limu) no ICT-positive children were found, while in the other two districts (Benatsemay and Dugdadewa) six ICT-positive children were found in each district.

**Table 4:** Confirmatory mapping results from four districts in Ethiopia declared endemic by original WHO mapping in 2013.

Region	District (woreda)	2013 WHO Mapping Results		Survey Design^§^	Schools Sampled	Children Tested	Schools Received Ivermectin*	ICT Positive (%)	Design Effect^†^	Survey Result
		# tested (site 1/site2)	# positive site1/site2 (% site 1/% site 2)							
Oromia	Boneya Bushe	100/100	4/1 (4%/1%)	Systematic	20	294	Yes	0 (0%)	NA	Pass
Oromia	Dugdadewa	91/100	4/0 (4.4%/0%)	Cluster	29	458	Yes	6 (1.3%)	1.3	Fail
Oromia	Haro Limu	100/100	4/0 (4%/0%)	Systematic	29	213	No	0 (0%)	NA	Pass
SNNP	Bena Tsemay	107/101	8/4 (7.5%/4.0%)	Systematic	19	348	No	8 (2.3%)	NA	Fail

*At least two schools in the district reported distributing Ivermectin (IVM)

^**†**^The design effect estimates the degree to which the cluster survey design increases the sample variance, relative to a simple random sample; this calculation of the design effect does apply to systematic survey designs (“NA” = not applicable)

^§^A systematic survey design implies that all schools in the district were included in the sample and was employed in smaller districts (i.e. those with ≤40 schools total); under a cluster survey design only 30 schools were visited.

In Tanzania, the results from the standard WHO mapping strategy, conducted in parallel with the confirmatory mapping in the same 10 districts, are shown in Table 5. In each district, approximately 200 individuals were sampled, typically from two purposefully selected hamlets; however, in two of the districts the hamlets were so small that additional hamlets were added to reach the sample size, while in one district the first selected hamlet was so large that the sample size was met without adding a second hamlet. Two of the ten districts had at least one site with an ICT positive result (three ICT positives were found in Same District Council and one ICT positive in Siha District), both of which would qualify the districts for MDA according to WHO guidelines.

**Table 5:** Results from the standard WHO mapping protocol (adults >15 years in two villages per district) from same districts as the confirmatory mapping tool implementation in Tanzania in 2015.

Region	District	Survey Design	Sites Sampled	People Tested	% Female	Mean Age (SD)	ICT Positives (%)	Survey Result
Arusha	Meru	WHO Standard Protocol	4	208	30.30%	39 (16)	0 (0%)	Pass
Arusha	Monduli DC	WHO Standard Protocol	2	211	53.60%	40 (15)	0 (0%)	Pass
Kagera	Karangwe DC	WHO Standard Protocol	2	205	58.50%	45 (15)	0 (0%)	Pass
Kagera	Muleba DC	WHO Standard Protocol	2	197	42.60%	42 (19)	0 (0%)	Pass
Kilimanjaro	Moshi DC	WHO Standard Protocol	3	199	41.70%	39 (15)	0 (0%)	Pass
Kilimanjaro	Moshi MC	WHO Standard Protocol	2	193	42.00%	30 (15)	0 (0%)	Pass
Kilimanjaro	Same DC	WHO Standard Protocol	2	202	31.70%	37 (17)	3 (1.5%)	Fail
Kilimanjaro	Siha	WHO Standard Protocol	2	191	48.70%	45 (19)	1 (0.5%)	Fail
Mara	Musoma	WHO Standard Protocol	1	195	34.90%	39 (17)	0 (0%)	Pass
Simiyu	Bariadi	WHO Standard Protocol	2	197	50.30%	37 (17)	0 (0%)	Pass

## Discussion

While mapping is an essential first step for lymphatic filariasis elimination programs, the current approach can produce uncertain results in some districts, leading to challenges for program managers regarding the decision to implement MDA. Given how resource-intensive it is for country programs to conduct MDA, it is important to get this decision right. This paper describes a confirmatory mapping tool that overcomes many of the pitfalls of the current approach, allowing program implementers to feel more confident in their decision to start or forego MDA. The main advantages of this new tool include a sampling strategy that results in a geographically-representative and unbiased sample, while still maintaining the efficiency of school-based sampling; a critical cutoff that protects against the potential for a single false positive test to drive endemicity status; and the targeting of upper-level primary school students as opposed to adults, for whom the presence of filarial antigens are likely indicative of recent, and not historic, transmission.

It is important to emphasize that this confirmatory mapping tool is not meant as a replacement for the standard WHO approach, but rather as a complementary tool that can be used to confirm whether active transmission is likely to be present when the results from the standard approach are inconclusive (e.g., only one positive adult). The standard WHO mapping approach is quick, simple, inexpensive, and effective at identifying high-transmission areas in need of mass treatment and thus should remain the primary approach for ruling-in areas with active transmission. The confirmatory mapping tool is based on a null hypothesis that transmission is active and therefore works well as a second-stage tool to rule-out uncertain areas. As of the end of 2015, there were 971 endemic districts requiring MDA that have yet to start [2]. For some of these districts, the initial mapping exercise took place years ago and there may be a need for a more rigorous survey to determine whether MDA is still required.

Another potential use for the confirmatory mapping tool is in post-endemic countries that have never undergone MDA but are ready to validate elimination. This may happen in areas where there is historical evidence of transmission but better infrastructure, the use of insecticide-treated nets (a secondary benefit of the malaria control programs), decreased contact with vectors through a reduction in breeding sites, or historical misdiagnosis of filarial morbidity mean that the country is no longer endemic. For these countries, as well as regions within endemic countries that have never received MDA and for which endemicity status is considered uncertain, the confirmatory mapping tool can provide greater confidence that no detectable foci of on-going transmission remain.

While the confirmatory mapping tool is more expensive and resource-intensive than the standard WHO approach, a cost analysis from Ethiopia and Tanzania suggests that when the long-term implications are considered, it is highly cost-effective when compared to unnecessary MDA. Specifically, remapping 55 districts in Ethiopia and Tanzania cost $450,000, but it avoided the unnecessary treatment of 8.1 million people across 52 districts, saving over $10,000,000. For NTD programs, the ability to avoid unnecessary MDA not only saves precious financial resources, it also means more time, energy and human capital can be dedicated to the areas where it is needed the most.

But does the confirmatory mapping tool always lead to the correct MDA decision? Unfortunately, the global LF elimination program does not have the luxury of time required for a long-term, empirical validation of the tool, which would require waiting at least five years to observe the persistence of low-level transmission or an emergence. Simulations suggest that geographically representative samples with many clusters are better able to detect transmission where it is low or focal [14]; however, short of sampling everywhere, no sampling strategy will be 100% accurate. The two comparison studies in Ethiopia and Tanzania suggest the confirmatory mapping tool performs well but it is not possible to conclude that the results are ‘validated.’ In Ethiopia, where four districts had been declared endemic by the WHO mapping approach when remapped using the confirmatory mapping tool, the tool classified two as not needing MDA. Interestingly, further investigation revealed that at least one of these districts had received treatment with ivermectin since the initial mapping was performed. In Tanzania, where the standard WHO approach was implemented at the same time as the confirmatory mapping tool, two of the ten districts were considered in need of MDA by the WHO mapping approach, compared with none of the ten districts requiring MDA by the confirmatory mapping tool. In one of these districts where the conclusions differed, only one positive adult was found by the WHO approach, calling into question the true need for MDA. The 2015 results of the WHO approach differ from the original WHO mapping results implemented in 1999–2004, as eight of the 10 districts were found to be non-endemic upon reexamination. Potential reasons for this difference include the increasingly widespread use of insecticide-treated nets for malaria, which target the same vector species as LF; a natural decline in infection intensity over time, perhaps aided by infrastructure improvements, in an area that was low to begin with; and chance, due to the imprecise nature of sampling from only two sentinel sites.

It is important to point out that the thresholds used for the WHO and confirmatory mapping approaches are different. According to the WHO mapping approach, a district fails if the point estimate of prevalence in a small convenience sample of adults is ≥1% and passes if it is <1%, while the threshold of confirmatory mapping is 2%. This latter figure may be somewhat misleading, however, because the critical cutoff for the confirmatory mapping approach requires there be a <6% chance that the true prevalence is ≥2%, which means the point estimate for the critical cutoff is actually <1%. The bigger question is whether either of these thresholds is correct. The WHO mapping threshold of 1% is based on the Chinese experience with microfilaremia in post-MDA settings, whereby communities with 1% MF at the time MDA was stopped tended to see infection continue to zero in the absence of MDA [15]. While this is based on empiric evidence, significant extrapolation is required to assume that the same threshold applies to pre-MDA settings. The threshold of <2% antigenemia, used in this confirmatory mapping tool, is taken directly from the LF transmission assessment survey guidelines and was chosen to serve as a conservative proxy for a prevalence of <1% MF [13]. Here again, the same concern with extrapolating this threshold to a pre-MDA setting applies. In short, there is no empiric evidence, of which we are aware, that directly addresses the optimal threshold for basing mapping decisions.

While the confirmatory mapping approach utilizes the efficiency of school-based sampling, the limitation of using the school platform is that it systematically excludes children who do not attend school. A study examining the difference in antigenemia among school-attending and non-attending children failed to find any significant difference, but this was in the context of an LF transmission assessment survey in Burkina Faso [16]. Because school attendance often declines with age, it may be important to investigate whether antigenemia is associated with school attendance among older school-age children in treatment naïve settings. Nonetheless, it is important to recognize that, for making MDA stopping decisions, WHO considers school-attending children to be sufficiently representative of all similarly aged children in settings where school attendance is at least 75% [13].

Finally, the utility of the confirmatory mapping tool has the potential to extend beyond LF to other diseases. In fact, this survey tool can be modified to test virtually any threshold, extending its value to other validation exercises. An immediate opportunity for extension of this tool is for onchocerciasis. With the onchocerciasis program target moving from control to elimination, areas that were categorized as hypo-endemic or of unknown endemicity, and consequentially left untreated, now require mapping to determine if MDA is necessary. A similar two-stage approach, whereby purposeful sampling is used to quickly and efficiently rule-in those areas in need of treatment and a confirmatory mapping tool is used in uncertain areas to rule-out those areas where MDA is not necessary, may provide a more robust framework for decision-making in onchocerciasis, as well as greater cohesiveness and clarity across PC NTD programs.

The confirmatory mapping tool represents an important addition to the monitoring and evaluation toolkit for program managers. In low-prevalence settings, this tool may enable program managers to make treatment decisions in districts previously blocked by inconclusive results or poor data. It has the potential to save time, money, resources and avoid unnecessary treatments, and it may provide sufficient evidence for programs in some areas to proceed from mapping directly to validation of elimination as a public health problem. With the 2020 elimination targets on the horizon, the confirmatory mapping tool may prove to be particularly useful for ‘shrinking the map’ and conserving resources for use in areas where they are needed most.

## Supporting information

S1 Appendix A detailed description of the cost-effectiveness analysis. (DOCX)

S1 Data Ethiopian confirmatory mapping data. (CSV)

S2 Data Tanzanian confirmatory mapping data. (CSV)

S3 Data Tanzanian WHO mapping data. (CSV)

## References

[1] The Lancet. Neglected tropical diseases: becoming less neglected. Lancet (London, England) [Internet]. 2014 4 12 [cited 2017 Apr 13];383(9925):1269 Available from: http://linkinghub.elsevier.com/retrieve/pii/S0140673614606292 10.1016/S0140-6736(14)60629-2 24725560

[2] World Health Organization. Global programme to eliminate lymphatic filariasis: progress report, 2015. Wkly Epidemiol Rec Relev épidémiologique Hebd [Internet]. 2016;39(91):441–60. Available from: http://www.who.int/wer/2015/wer9038.pdf?ua=1%5Cnhttp://www.who.int/wer

[3] Bockarie MJ, Rebollo MP. Reducing the population requiring interventions against lymphatic filariasis in Africa. Lancet Glob Heal [Internet]. 2016 3 [cited 2017 Apr 13];4(3):e154–5. Available from: http://linkinghub.elsevier.com/retrieve/pii/S2214109X15002922 10.1016/S2214-109X(15)00292-2 26874545

[4] World Health Organization. Operational guidelines for rapid mapping of Bancroftian filariasis in Africa. 2000;WHO/CDS/CP.

[5] World Health Organization. Report of the WHO Strategic and Technical Advisory Group for Neglected Tropical Diseases, April 21–22, 2015 [Internet]. Geneva; 2015. Available from: http://www.who.int/neglected_diseases/NTD_STAG_report_2015.pdf?ua=1

[6] World Health Organization. Preventive chemotherapy in human helminthiasis [Internet]. Geneva; 2006. Available from: http://apps.who.int/iris/bitstream/10665/43545/1/9241547103_eng.pdf

[7] Gass K, de Rochars MVEB, Boakye D, Bradley M, Fischer PU, Gyapong J, et al A multicenter evaluation of diagnostic tools to define endpoints for programs to eliminate bancroftian filariasis. PLoS Negl Trop Dis. 2012;6(1). 10.1371/journal.pntd.0001479 PMC3260316 22272369

[8] Wanji S, Amvongo-Adjia N, Koudou B, Njouendou AJ, Chounna Ndongmo PW, Kengne-Ouafo JA, et al Cross-Reactivity of Filariais ICT Cards in Areas of Contrasting Endemicity of Loa loa and Mansonella perstans in Cameroon: Implications for Shrinking of the Lymphatic Filariasis Map in the Central African Region. Bottomley C, editor. PLoS Negl Trop Dis [Internet]. 2015 11 6 [cited 2017 Apr 13];9(11):e0004184 Available from: http://www.ncbi.nlm.nih.gov/pubmed/26544042 doi: 10.1371/journal.pntd.0004184 26544042 10.1371/journal.pntd.0004184 PMC4636288

[9] Chu BK, Deming M, Biritwum NK, Bougma WR, Dorkenoo AM, El-Setouhy M, et al Transmission Assessment Surveys (TAS) to Define Endpoints for Lymphatic Filariasis Mass Drug Administration: A Multicenter Evaluation. PLoS Negl Trop Dis. 2013;7(12). 10.1371/journal.pntd.0002584 PMC3855047 24340120

[10] Drexler N, Washington CH, Lovegrove M, Grady C, Milord MD, Streit T, et al Secondary Mapping of Lymphatic Filariasis in Haiti-Definition of Transmission Foci in Low-Prevalence Settings. Ghedin E, editor. PLoS Negl Trop Dis [Internet]. 2012 10 11 [cited 2017 Apr 18];6(10):e1807 Available from: http://www.ncbi.nlm.nih.gov/pubmed/23071849 doi: 10.1371/journal.pntd.0001807 23071849 10.1371/journal.pntd.0001807 PMC3469481

[11] Witt C, Ottesen E a. Lymphatic filariasis: an infection of childhood. Trop Med Int Heal. 2001;6(8):582–606. 10.1046/j.1365-3156.2001.00765.x 11555425

[12] P. Rebollo M, Sime H, Assefa A, Cano J, Deribe K, Gonzalez-Escalada A, et al Shrinking the Lymphatic Filariasis Map of Ethiopia: Reassessing the Population at Risk through Nationwide Mapping. Bethony JM, editor. PLoS Negl Trop Dis [Internet]. Public Library of Science; 2015 11 5 [cited 2016 Jun 30];9(11):e0004172 Available from: http://dx.plos.org/10.1371/journal.pntd.0004172 doi: 10.1371/journal.pntd.0004172 26539700 10.1371/journal.pntd.0004172 PMC4634982

[13] World Health Organization. Monitoring and epidemiological assessment of mass drug administration in the global programme to eliminate lymphatic filariasis: a manual for national elimination programmes. 2011;WHO/HTM/NT Available from: http://www.who.int/lymphatic_filariasis/resources/9789241501484/en/

[14] Harris JR, Wiegand RE, Sodahlon Y, Fasuyi O, Mathieu E, Faris R. Detecting infection hotspots: Modeling the surveillance challenge for elimination of lymphatic filariasis. Basáñez M-G, editor. PLoS Negl Trop Dis [Internet]. R Foundation for Statistical Computing; 2017 5 19 [cited 2017 May 26];11(5):e0005610 Available from: http://dx.plos.org/10.1371/journal.pntd.0005610 doi: 10.1371/journal.pntd.0005610 28542274 10.1371/journal.pntd.0005610 PMC5453617

[15] World Health Organization. Control of Lymphatif Filariasis in China. Manila: World Health Organization’s Regional Office for the Western Pacific; 2003 126–133 p.

[16] Gass K, Tiendrebeogo R, Bougma WR, Sidibe S, Kyelem D. Can school-based sampling replace community-based sampling to measure circulating filarial antigen in areas where school attendance is low? Am Soc Trop Med Hyg. 2011;Conference.

