# Supplementary material for: The rationale and cost-effectiveness of a confirmatory mapping tool for lymphatic filariasis: Examples from Ethiopia and Tanzania
Source: PLoS Negl Trop Dis. 2017 Oct 4;11(10):e0005944. doi: 10.1371/journal.pntd.0005944 (PMC5643143; doi:10.1371/journal.pntd.0005944)
Supplement: S1 Appendix — (DOCX) [file pntd.0005944.s001.docx]

**S1 Appendix. A detailed description of the cost-effectiveness analysis.**

To calculate the cost-effectiveness of the confirmatory mapping, we subtracted the actual costs of confirmatory mapping in all districts from the costs averted by avoiding treatment of districts found by confirmatory mapping to be non-endemic for LF. Actual costs of confirmatory mapping were available in the financial records of the Neglected Tropical Disease Support Center. In accordance with current WHO guidelines for elimination of LF, costs of eliminating LF in a district include, at minimum, five rounds of MDA at 65% coverage, two sentinel and spot check assessments, and three transmission assessment surveys [1]. Because treatment costs vary according to district population, we estimated district population using a database maintained by the WHO AFRO Region. The costs of the three components were estimated as follows:

1. Five rounds of MDA: Financial costs for MDA were calculated to include the actual monetary cost to the country program for each dose [2]. In 2007, Goldman et al reported the financial and economic costs per person for a number of countries [2]. Because the costs for Tanzania were reported in this paper, when calculating cost savings for Tanzania, we used the average financial and economic costs per dose for Tanzania. This worked out to $0.37 per dose. Because specific costs from Ethiopia were not included in this paper, when calculating the cost savings for Ethiopia, we used the average financial costs per dose for all rounds of MDA in the African region recorded in the paper. This worked out to $0.26 per dose.
2. Two sentinel and spot check assessments: According to WHO recommendations, each sentinel site assessment and each spot check site assessment includes testing 300 people with either filariasis test strip (FTS) or immunochromatographic card tests (ICT) [1]. Sentinel and spot check assessments should be conducted both at baseline and after five rounds of MDA. An assessment after three rounds of MDA is also recommended but not required; therefore, it is not included. We assumed that sentinel and spot check site testing would be conducted using the filariasis test strip (FTS), which we estimated to cost $1.80 per test including shipping. We also estimated that an additional $1,000 of expenses for personnel, supplies, transportation, and training would be required for each round of sentinel site/spot check assessments. Therefore, the cost per district of two sentinel site assessments and two spot check assessments was estimated at $4,160.
3. Three transmission assessment surveys (TAS): A recent review of TAS supported by the United State Agency for International Development (USAID) and conducted in 2012-2014 found the median cost of a TAS in the Africa region was $23,305.50 per evaluation unit (EU) [3]. The same review found that a median of 2.13 implementation units (IUs) or districts were included in each evaluation unit (EU). Assuming that each EU included an average of 2.125 districts, the cost per district was estimated at $10,967.29 per TAS, or $32,901.88 for three TAS.

**References**

1. World Health Organization. Monitoring and epidemiological assessment of mass drug administration in the global programme to eliminate lymphatic filariasis: a manual for national elimination programmes. 2011;WHO/HTM/NT. Available from: http://www.who.int/lymphatic_filariasis/resources/9789241501484/en/

2. Goldman AS, Guisinger VH, Aikins M, Amarillo MLE, Belizario VY, Garshong B, et al. National Mass Drug Administration Costs for Lymphatic Filariasis Elimination. King C, editor. PLoS Negl Trop Dis [Internet]. WHO; 2007 Oct 31 [cited 2017 May 26];1(1):e67. Available from: http://dx.plos.org/10.1371/journal.pntd.0000067

3. Brady MA, Stelmach R, Davide-Smith M, Johnson J, Pou B, Koroma J, et al. Costs of Transmission Assessment Surveys to Provide Evidence for the Elimination of Lymphatic Filariasis. Lammie PJ, editor. PLoS Negl Trop Dis [Internet]. World Health; 2017 Feb 1 [cited 2017 Apr 13];11(2):e0005097. Available from: http://dx.plos.org/10.1371/journal.pntd.0005097
